# Global Research Landscapes in Keloid Treatment: A Bibliometric and Visual Analysis

**DOI:** 10.1111/jocd.71054

**Published:** 2026-07-23

**Authors:** Yilin Wang, Jintong Wu, Zijie Tang, Rui Wang, Chengxin Li

**Affiliations:** ^1^ Department of Dermatology 1st Medical Center of Chinese PLA General Hospital Beijing China; ^2^ State Key Laboratory of Kidney Diseases 1st Medical Center of Chinese PLA General Hospital Beijing China

**Keywords:** bibliometrics, CiteSpace, keloid, treatment, VOSviewer

## Abstract

**Background:**

Keloid treatment is a pressing challenge in clinical practice. We aim to comprehensively summarize the knowledge structure, research topics, and research trends.

**Methods:**

This study retrieved articles published between 1995 and 2025 from the Science Citation Index Expanded within the Web of Science Core Collection. Additionally, clinical trials indexed in PubMed were selected to evaluate progress in clinical research. Using bibliometric analysis tools and methods, such as VOSviewer, Scimago Graphica, and CiteSpace, we systematically explored the current research landscape.

**Results:**

China has the most significant number of articles, and the Chinese Academy of Medical Sciences and Peking Union Medical College are the most productive institutions. Bayat, A, is the most prolific author. Co‐citation clustering analysis identified research clusters encompassing molecular pathway analysis, comprehensive management and clinical guidelines, pathogenesis and fibroblast research, genetic susceptibility, synergistic drug delivery, photodynamic therapy, and novel compound therapies. Keyword burst analysis highlights the field's comprehensive advancement toward precision targeting, evidence‐based medicine, and renewed understanding of disease essence.

**Conclusions:**

Overall, this study indicates that keloid treatment focuses on precision targeting and systemic management, with frontiers expanding to delivery systems, molecular biomarkers, extracellular vesicles, metabolic reprogramming, and evidence‐based management centered on guidelines and quality of life. Novel and combined treatment approaches require further exploration.

## 1 Introduction

Keloid is a common fibrotic skin disorder characterized by excessive growth beyond the original wound margins and a long‐term persistence without a quiescent or regressive phase [1]. The global prevalence ranges from approximately 0.09% to 16%, with significant variation across racial/genetic groups. Individuals of African descent face the highest risk, followed by Asians, while Caucasians have the lowest incidence [2]. Statistics indicate that approximately 100 million new scars form each year globally, with 11 million potentially progressing to keloids [3]. Although keloids are non‐metastatic, they exhibit tumor‐like characteristics and share numerous similarities with tumors [4]. Keloid scars frequently cause pain or itching, potentially leading to severe functional impairment and diminished quality of life (QoL), thereby increasing the burden on both patients and healthcare systems [5]. The primary treatment objectives include improving scar appearance, alleviating symptoms, and enhancing patients' QoL. Multiple treatment approaches currently exist, including non‐pharmacological and pharmacological therapies, as well as novel materials [6].

However, the microenvironment, pathogenesis, and development of keloids are highly complex, limiting the efficacy of various treatment modalities [7]. For instance, intralesional corticosteroid injections yield inconsistent results and carry side effects such as skin atrophy and telangiectasia, fundamentally due to uncontrolled drug distribution within tissues and transient high‐concentration release [6, 8]. This clinical challenge has directly driven the development of novel drug delivery systems to prolong drug action, enhance bioavailability, and minimize side effects [9, 10]. Furthermore, existing therapies fail to precisely target the abnormal fibrotic signaling pathways characteristic of keloids [11]. Therefore, current research focuses on fundamentally regulating the pathological microenvironment of keloids to achieve greater efficacy with fewer systemic side effects. Collectively, these research directions signal a transition in keloid treatment from symptom management to causal intervention.

Bibliometrics, as an independent discipline, is widely used for literature analysis through quantitative analysis of existing research in a specific field [12, 13]. Currently, systematic quantitative integration and analysis of the vast literature on keloid treatment remain lacking. This study constructs a comprehensive data‐driven research landscape by screening the existing literature and applying bibliometric methods. Utilizing multiple tools to map keloid treatment applications provides an overview and visual representation of this rapidly evolving frontier field. By identifying core researchers and tracking thematic evolution, it empowers clinicians and researchers to gain clearer insights into the global status, research hotspots, and emerging directions within this domain.

## 2 Materials and Methods

### 2.1 Data Sources and Search Strategies

We conducted a comprehensive literature search on keloid treatment in the Science Citation Index Expanded (SCI‐E) of the Web of Science Core Collection (WoSCC) database. The literature types included articles and review articles, with the language restricted to English. To enhance the scientific rigor of this study and avoid the limitations of relying on a single database, clinical trials indexed in PubMed were also searched as a supplementary data source. The search was conducted on November 30, 2025, covering the time span from January 1995 to 2025. The search and data download for all publications were completed within 1 day to prevent bias from frequent database updates. The specific search terms and strategy are provided in Supporting Information.

### 2.2 Data Acquisition and Visualized Analysis

**FIGURE 1:**
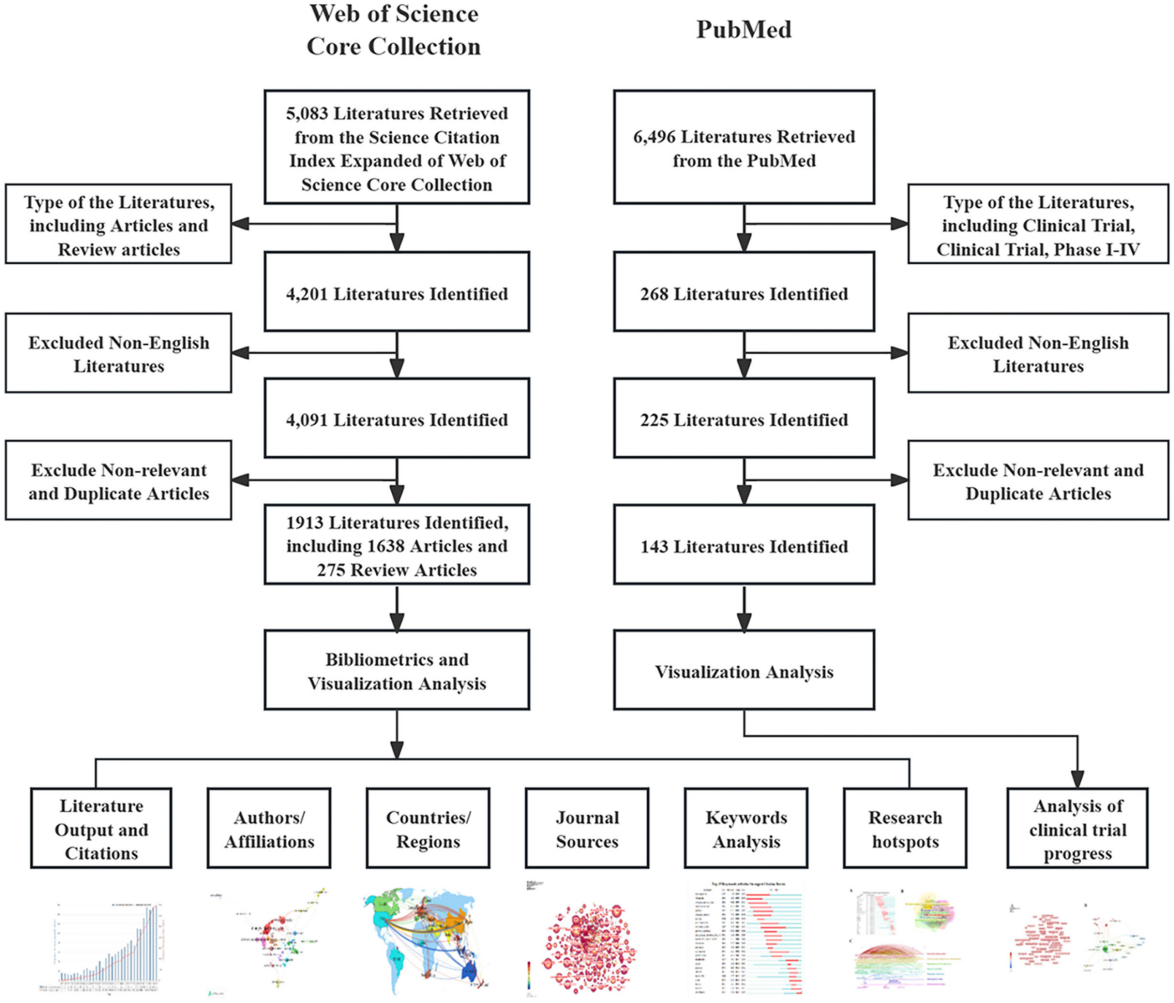
Screening flowchart of keloid treatment from the perspective of bibliometric analysis.

To ensure the retrieved publications were highly relevant to the main topic, two researchers screened and recorded all publications by reading their titles and abstracts. Ultimately, 1913 articles from the WoSCC met the inclusion criteria for this study, comprising 1638 articles and 275 review articles. Publications meeting the inclusion criteria were exported as plain‐text files in the “Full Record and Cited References” format. A total of 143 articles sourced from the PubMed database were utilized for clinical progress analysis. Then, we employed bibliometric and visualization tools, namely WoSCC, VOSviewer (version 1.6.18), CiteSpace (version 6.3.R1), and Scimago Graphica (version 1.0.50), to analyze publications, countries/regions, institutions, authors, journals, references, keywords, and citations. The WoSCC was used to analyze the number of annual publications, annual time cited, and developmental trends across different years. VOSviewer is a visualization tool that constructs association networks among countries/regions and institutions based on collaborative or co‐occurrence data [14]. Scimago Graphica excels at transforming analyzed network data into more interactive visualizations. CiteSpace, a practical visual software tool, captures developmental processes and trends within research fields, particularly in literature citations and keyword bursts [15]. Beyond conventional bibliometric data, this study introduces the Hirsch index (H‐index) to provide a more comprehensive evaluation [16, 17]. The use of those bibliometric data can assess an author's scholarly output and its impact on citations within the academic community, helping readers identify more promising researchers and capture research frontiers and trends [16]. The detailed flow chart is shown in Figure 1.

## 3 Results

### 3.1 Quantity and Trends Analysis of Published Papers

**FIGURE 2:**
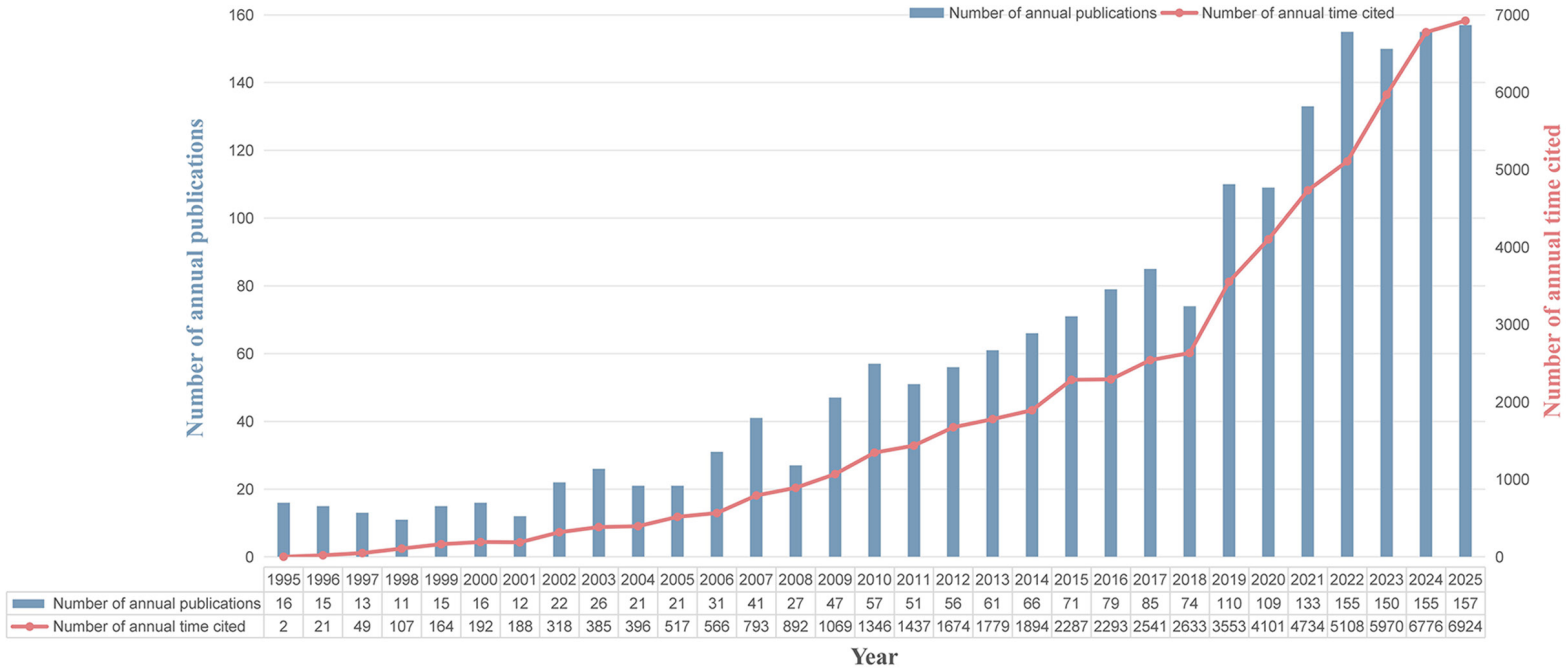
Visualization of annual publication trends and aggregate citation counts for articles. The blue bar graph indicates annual publications, while the pink line graph depicts the annual time cited.

We retrieved 1913 articles from the SCI‐E of WoSCC. As illustrated in Figure 2, both the number of papers and citation frequency rose steadily from 1995 to 2025. This indicates a significant increase in research output, citation impact, and scholarly attention in keloid treatment research, indicating this field as a worthy academic hotspot for in‐depth exploration.

### 3.2 Analysis of Collaborating Countries/ Regions and Institutions

**FIGURE 3:**
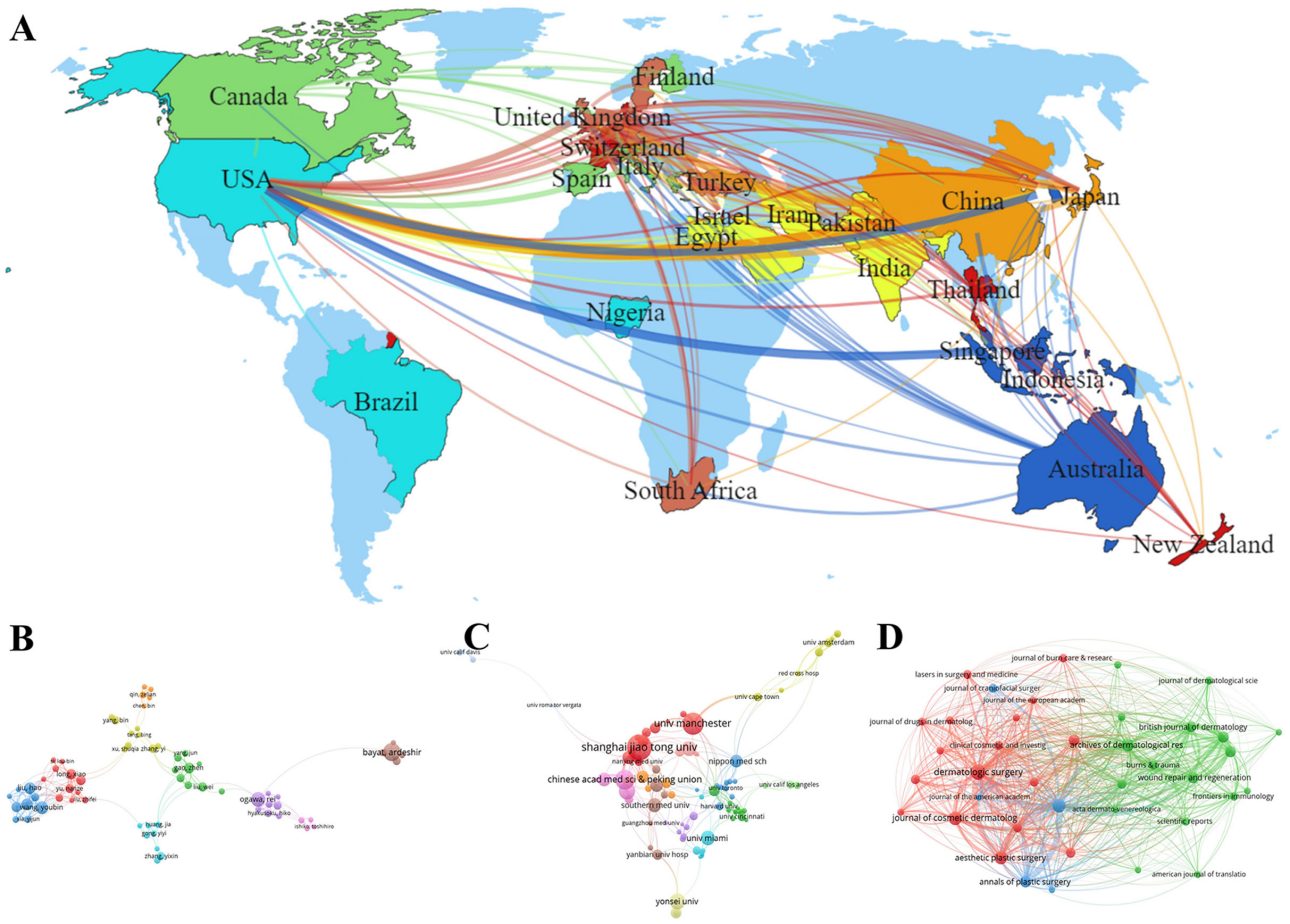
Analysis of collaborative efforts among countries/regions (A), authors (B), institutions (C), and journals (D). Collaboration network: Circle sizes in the figure denote the publishing output, and connecting lines between nodes represent the collaboration.

The 1913 identified articles were published in 72 countries. As shown in Figure 3A, only countries with at least 5 publications are displayed. Table 1 highlights the major research countries in keloid treatment, including China, the United States, and South Korea. Figure 3C lists the productive institutions with at least 5 publications, which shows that within‐country/region collaborative networks were particularly intensive. Table 2 lists the top 10 most productive institutions. Six of the top 10 most productive institutions were from China, indicating that Chinese institutions made substantial contributions to research output in keloid treatment.

**TABLE 1:** The top 10 productive countries.

Rank	Countries	Article counts	Total citations	Average citations per article	Total link strength
1	China	707	11 666	16.50	81
2	USA	327	17 507	53.54	116
3	South Korea	137	3308	24.15	39
4	England	116	6401	55.18	78
5	Japan	101	5606	55.50	46
6	Germany	86	5799	67.43	77
7	Egypt	56	1165	20.80	9
8	Italy	51	1757	34.45	28
9	Netherlands	49	3026	61.76	33
10	Singapore	43	1834	42.65	20

**TABLE 2:** The top 10 institutions by publication volume.

Rank	Affiliations	Countries	Article counts	Total citations	Average citations per article
1	Chinese Academy of Medical Sciences and Peking Union Medical College	China	117	1637	13.99
2	Shanghai Jiao Tong University	China	77	1599	20.77
3	The University of Manchester	England	61	3771	61.82
4	Yonsei University	South Korea	39	688	17.64
5	National University of Singapore	Singapore	36	729	20.25
6	University of Miami	America	33	1496	45.33
7	Southern Medical University	China	31	2514	81.10
8	Sichuan University	China	29	280	9.66
9	Fudan University	China	26	355	13.65
10	Sun Yat‐sen University	China	26	384	14.77

### 3.3 Analysis of Co‐Authorship Networks and Core Author Distribution

Over the past 30 years, 8082 authors contributed to the 1913 identified articles. Figure 3B visualizes the collaborative relationships among authors who have published at least five papers and share collaborative ties. Table 3 lists the countries and the total number of citations for these authors. The most published author in this field was Bayat, A from the University of Cape Town, South Africa, with 45 publications and 1937 citations. The author with the highest average number of times cited per item is Ogawa, R., from Nippon Medical School in Japan. Most prolific authors were from highly productive countries or institutions and showed strong collaborative relationships.

**TABLE 3:** The top 10 authors with the highest publication volume.

Rank	Author	Documents	H‐index	Citations	Average times cited per item	Countries	Institutions
1	Bayat [18]	45	59	1937	43.04	South Africa	University of Cape Town/The University of Manchester
2	Ogawa [19]	30	51	2744	91.47	Japan	Nippon Medical School/Southern Medical University
3	Park [20]	25	17	387	15.48	South Korea	Yonsei University
4	Wang [21]	23	17	345	15	China	Chinese Academy of Medical Sciences—Peking Union Medical College
5	Lim [22]	21	26	476	22.67	Singapore	National University of Singapore
6	Niessen [23]	20	38	1267	63.35	Netherlands	VU University Medical Center Amsterdam
7	Hao [24]	19	10	231	12.16	China	Chinese Academy of Medical Sciences—Peking Union Medical College
8	Liu [25]	19	12	243	12.79	China	Chinese Academy of Medical Sciences—Peking Union Medical College
9	Jin [26]	18	19	145	8.06	China	Yanbian University
10	Lee [27]	17	88	430	25.29	South Korea	Seoul National University Bundang Hospital and Seoul National University College of Medicine

### 3.4 Analysis of Journals

**FIGURE 4:**
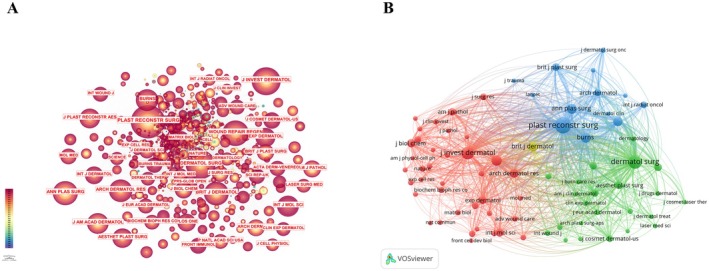
Analysis of co‐cited journals in research. (A) Visualization of the co‐cited journal network. (B) Visualization of the cited journal network, with circle size denoting the total link strength (TLS) of the documents represented by the nodes.

We analyzed the 1913 included publications from 486 journals using Citespace and VOSviewer (Figure 3D), and the top 10 journals by total citations are shown in Table 4. The leading journals include *Plastic and Reconstructive Surgery* (58 publications, total 4917 citations), *Journal of Investigative Dermatology* (38 publications, total 2529 citations), and *Dermatologic Surgery* (81 publications, total 4142 citations). The co‐cited journal network, as shown in Figure 4A,B, is visualized, with circle size denoting the total link strength of the documents represented by the nodes.

**TABLE 4:** The top 10 journals.

Rank	Journal	Count	IF	JCR	Total citations	Average times cited per item
1	*Dermatologic Surgery*	81	2.2	Q2	4142	51.14
2	*Journal of Cosmetic Dermatology*	60	2.5	Q2	910	15.17
3	*Plastic and Reconstructive Surgery*	58	3.4	Q1	4917	84.78
4	*Archives of Dermatological Research*	55	2.1	Q3	1856	337.75
5	*Aesthetic Plastic Surgery*	54	2.8	Q1	1354	25.07
6	*Annals of Plastic Surgery*	43	1.6	Q3	1235	28.72
7	*Wound Repair and Regeneration*	39	3.4	Q1	1716	44
8	*Experimental Dermatology*	38	3.1	Q2	1295	34.08
9	*Burns*	38	2.9	Q1	1089	28.66
10	*Journal of Investigative Dermatology*	38	5.7	Q1	2529	66.55

### 3.5 Analysis of Co‐Citation and Clustered Network

**FIGURE 5:**
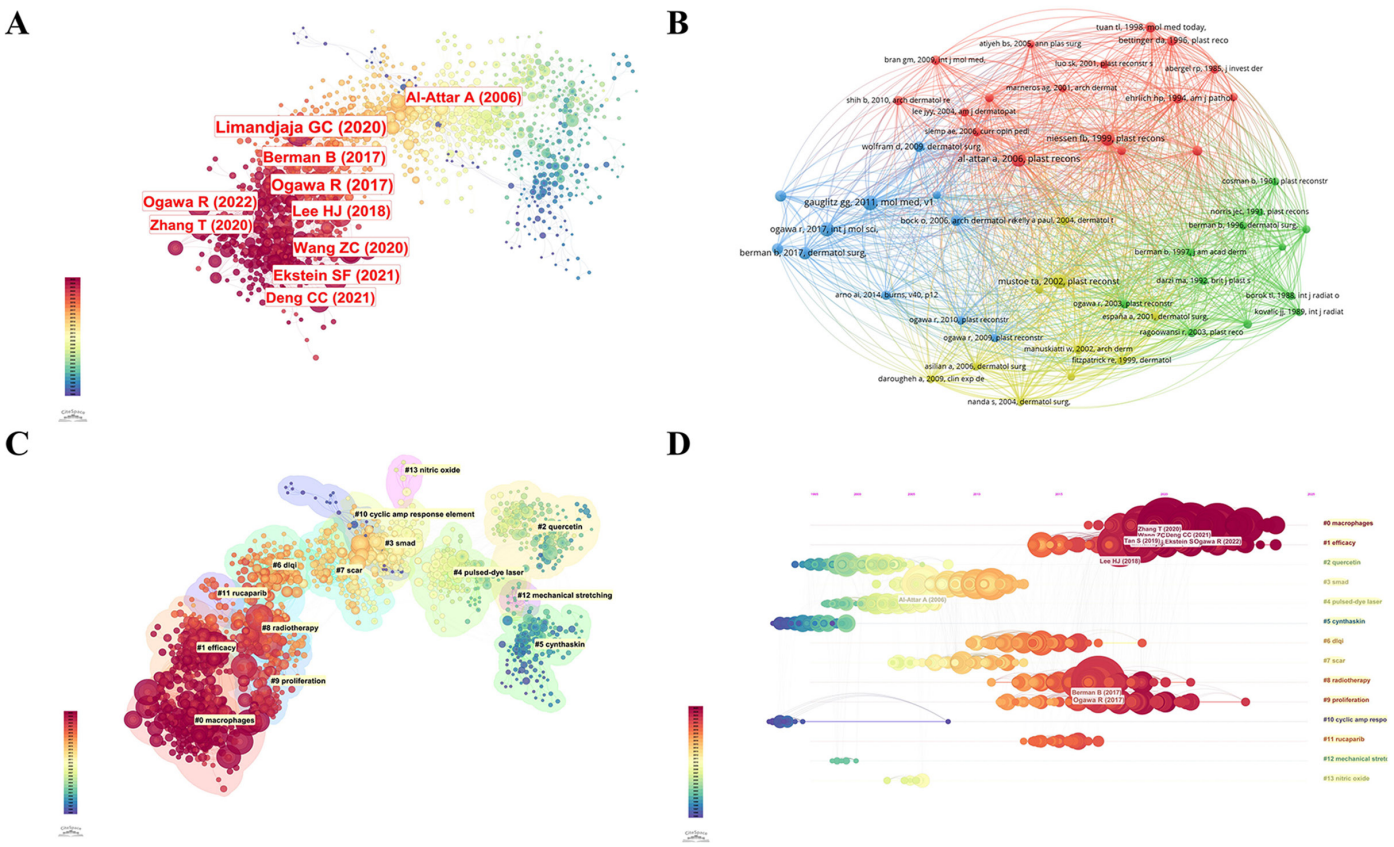
Analysis of references. (A) Visualization of the co‐cited reference network. (B) Top 50 co‐cited references, with circle size indicating the total link strength of the documents represented by the nodes. (C) Cluster diagrams representing co‐cited references. (D) Timeline view of the co‐cited reference cluster analysis. Connecting lines indicate co‐citation relationships between publications, and changes in node color represent temporal shifts.

**FIGURE 6:**
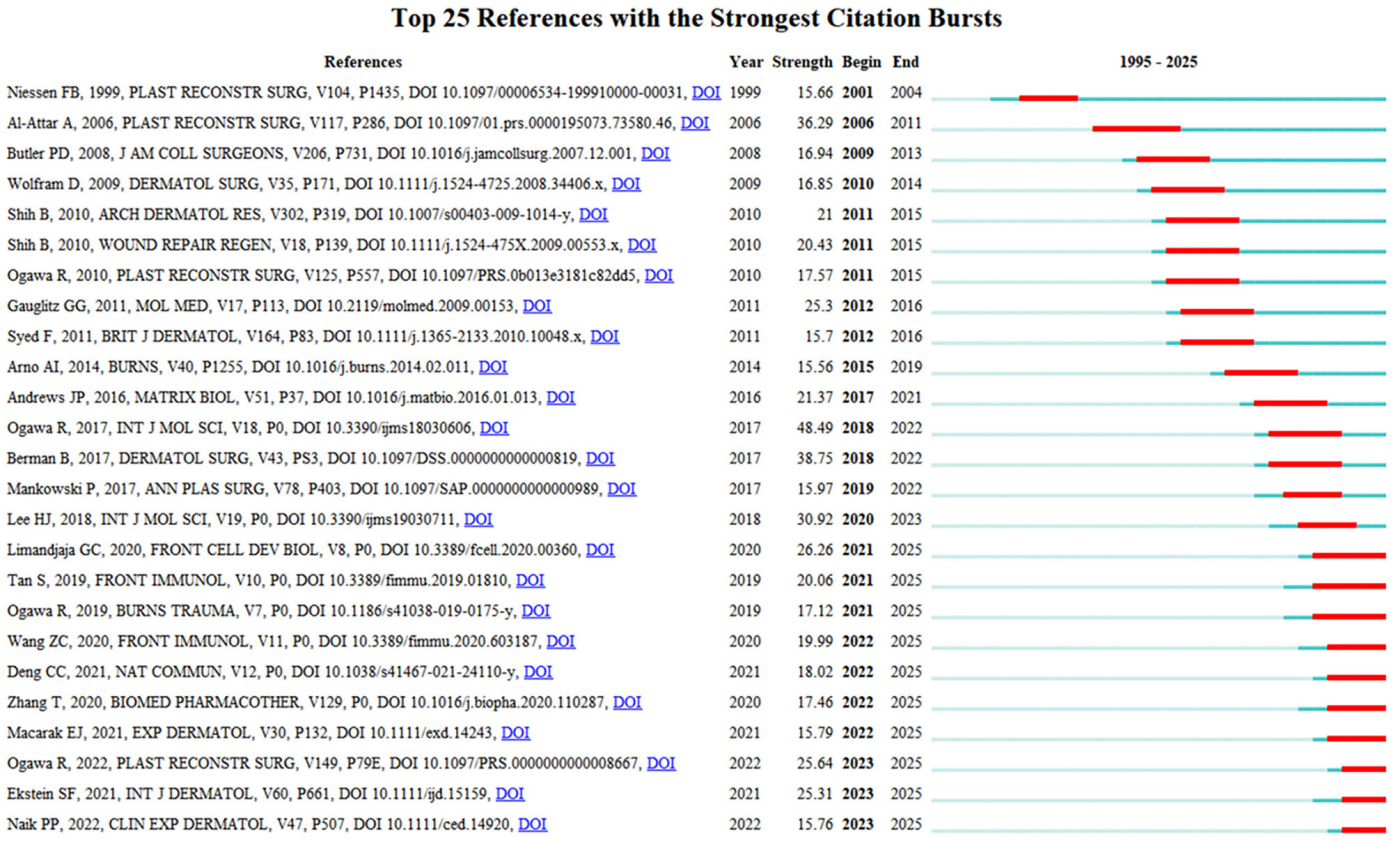
The top 25 references with the strongest citation bursts generated by CiteSpace.

The 1913 publications and 35 978 references were analyzed using CiteSpace. Tables 5 and 6 list the top 10 most‐cited and co‐cited references. Figure 5A identifies the co‐citation network of the literature, while Figure 5B showcases the top 50 articles according to citation frequency. Figure 5C presents a network of co‐cited literature clustered by thematic directions, indicating that researchers are committed to deepening the understanding of keloids at both the molecular mechanisms and physical factors levels, while evaluating and optimizing existing therapies. Concurrently, they are actively exploring novel drugs and biomaterials. The ultimate goal is to enhance treatment efficacy and improve patients' quality of life. Figure 5D displays co‐cited reference clustering on a timeline, offering insights into the evolution of node knowledge over time. Figure 6 shows the top 25 references with the strongest citation bursts.

**TABLE 5:** The top 10 highly cited articles.

Rank	Title	Journal	IF 2025	JCR 2025	Year	Citations
1	Hypertrophic scarring and keloids: pathomechanisms and current and emerging treatment strategies [28]	*Molecular Medicine*	6.4	Q1	2011	1060
2	Extracellular matrix reorganization during wound healing and its impact on abnormal scarring [29]	*Advances in Wound Care*	5.6	Q1	2015	1051
3	International clinical recommendations on scar management [30]	*Plastic and Reconstructive Surgery*	3.4	Q1	2002	740
4	Keloid and hypertrophic scars are the result of chronic inflammation in the reticular dermis [31]	*International Journal of Molecular Sciences*	4.9	Q1	2017	673
5	On the nature of hypertrophic scars and keloids: a review [32]	*Plastic and Reconstructive Surgery*	3.4	Q1	1999	636
6	Skin scarring [3]	*British Medical Journal*	43	Q1	2003	506
7	Keloids and hypertrophic scars: pathophysiology, classification, and treatment [33]	*Dermatologic Surgery*	2.2	Q2	2017	496
8	Hypertrophic scars and keloids‐a review of their pathophysiology, risk factors, and therapeutic management [34]	*Dermatologic Surgery*	2.2	Q2	2009	485
9	Keloid pathogenesis and treatment [35]	*Plastic and Reconstructive Surgery*	3.4	Q1	2006	450
10	Quality of life of patients with keloid and hypertrophic scarring [36]	*Archives of Dermatological Research*	2.1	Q3	2006	408

**TABLE 6:** The top 10 co‐cited references.

Author	Title	Journal	Year	Citations	Total link strength
Al‐Attar, A	Keloid pathogenesis and treatment [35]	*Plastic and reconstructive surgery*	2006	247	4298
Gauglitz, G	Hypertrophic scarring and keloids: pathomechanisms and current and emerging treatment strategies [28]	*Molecular medicine*	2011	247	3934
Ogawa, R	Keloid and hypertrophic scars are the result of chronic inflammation in the reticular dermis [31]	*International Journal of Molecular Sciences*	2017	225	3309
Niessen, FB	On the nature of hypertrophic scars and keloids: a review [32]	*Plastic and reconstructive surgery*	1999	223	4648
Mustoe, TA	International clinical recommendations on scar management [30]	*Plastic and reconstructive surgery*	2002	222	5262
Berman, B	Keloids and hypertrophic scars: pathophysiology, classification, and treatment [33]	*Dermatologic surgery*	2017	167	2290
Andrews, JP	Keloids: the paradigm of skin fibrosis—pathomechanisms and treatment [11]	*Matrix Biology*	2016	140	1729
Lee, HJ	Recent understandings of biology, prophylaxis and treatment strategies for hypertrophic scars and keloids [37]	*International Journal of Molecular Sciences*	2018	134	1840
Limandjaja, GC	The keloid disorder: heterogeneity, histopathology, mechanisms and models [23]	*Frontiers in Cell and Developmental Biology*	2020	128	1553
Wolfram, D	Hypertrophic scars and keloids‐a review of their pathophysiology, risk factors, and therapeutic management [34]	*Dermatologic Surgery*	2009	127	1880

### 3.6 Analysis of Research Trends and Burst Detection

**FIGURE 7:**
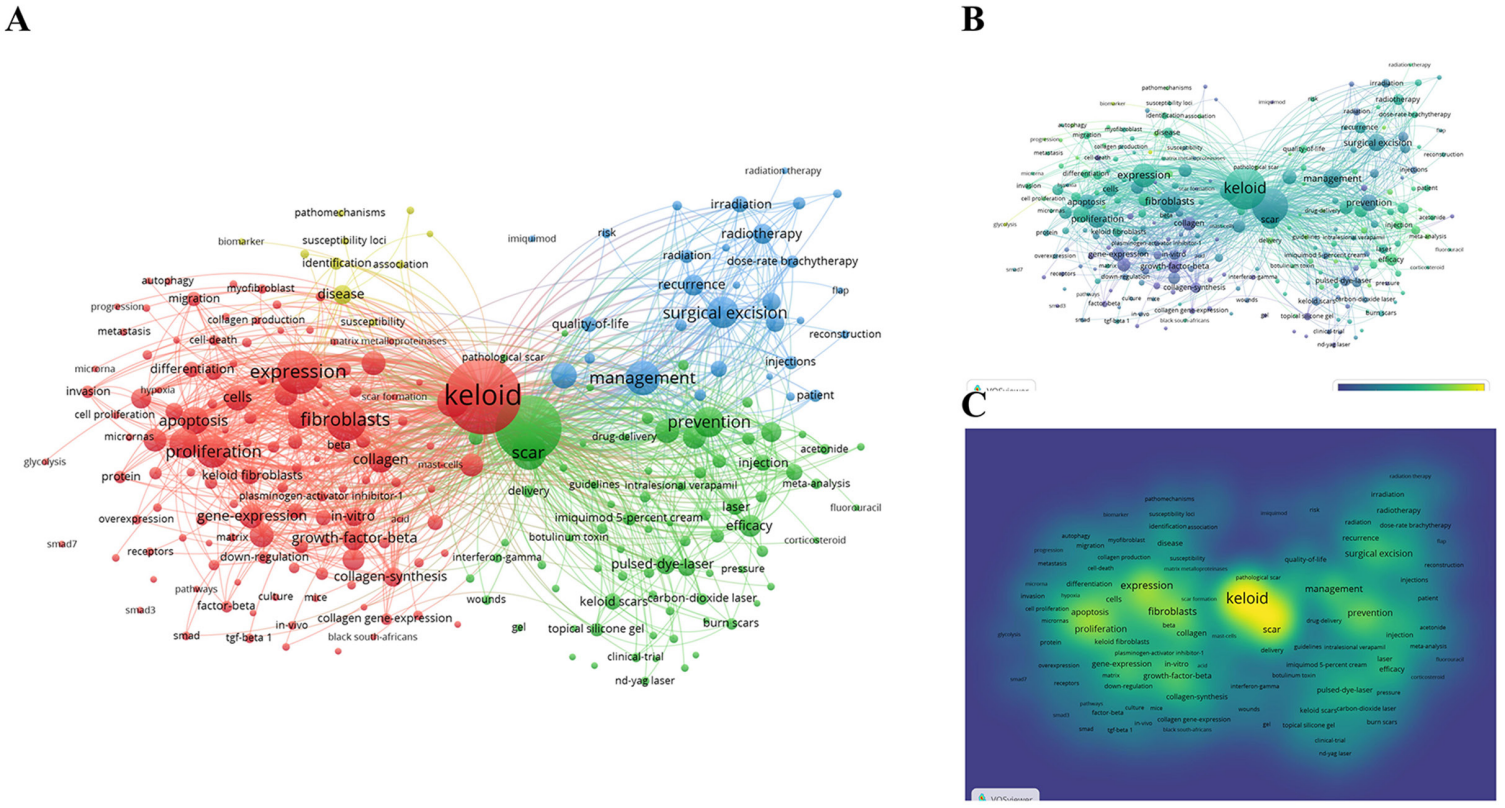
Visualization of the co‐occurrence of keywords (A), the keyword overlay (B), and keyword density (C) generated by VOSviewer software.

Figure 7A illustrates the co‐occurrence of keywords, while Figure 7B,C depict the keyword overlay and density generated by VOSviewer. Each circle represents a keyword, while the size of the circles indicates the frequency and research hotspots. Furthermore, Figure 7B reveals temporal shifts in keywords: Before 2015, keywords centered on pathology and physical intervention therapies, with a focus on collagen, gene expression, cell death mechanisms, and early physical/radiation therapies. From 2020 to 2025, keywords centered on precision targeting and systemic management, with frontiers expanding to novel delivery systems, molecular biomarkers, extracellular vesicles, metabolic reprogramming (glycolysis), and evidence‐based management centered on guidelines and quality of life.

**FIGURE 8:**
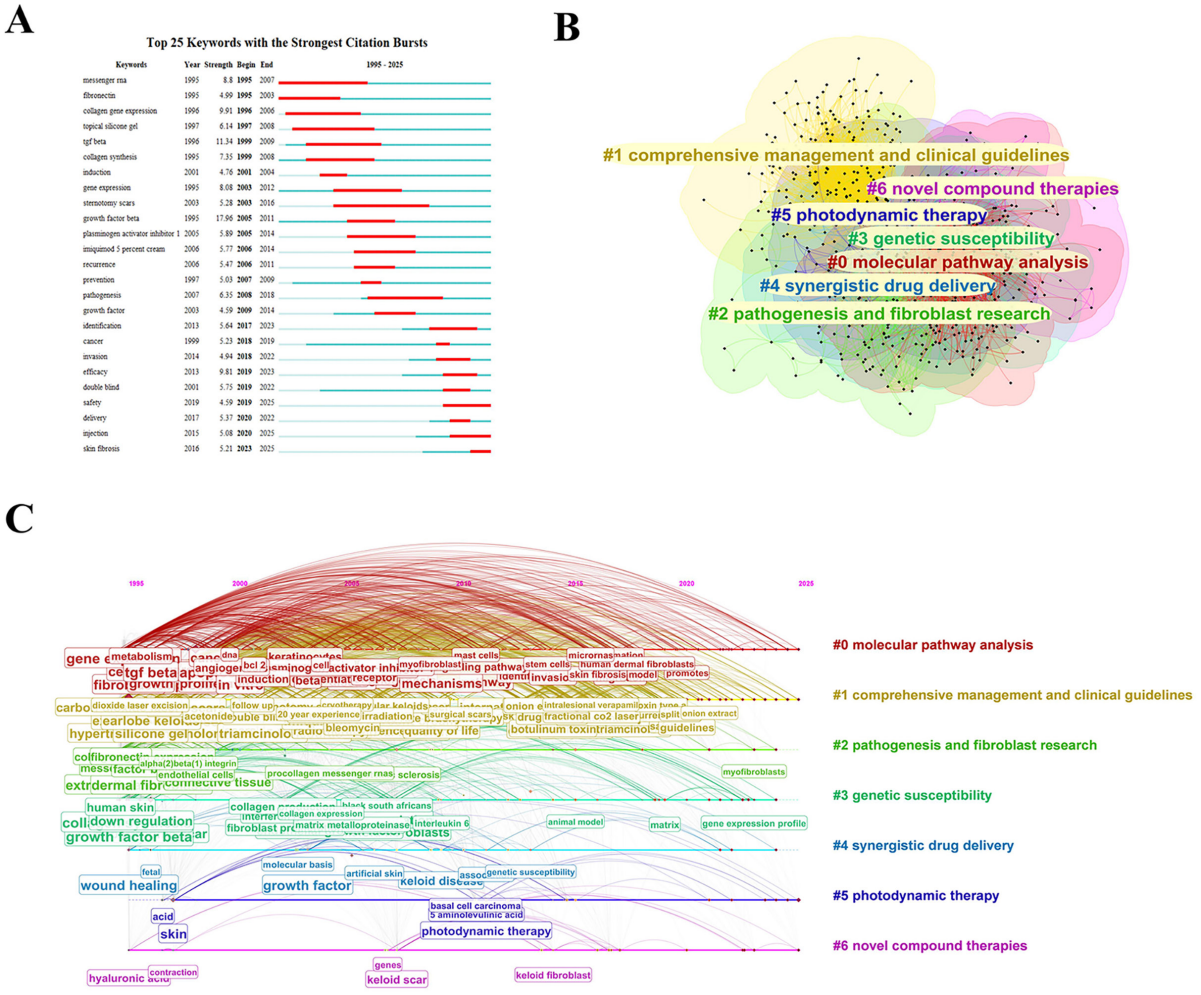
Analysis of keyword. (A) The 25 most significant keywords display substantial citation bursts. (B) Cluster diagrams of keywords. (C) Timeline view of keyword cluster analysis.

The top 25 high‐frequency keywords were generated by CiteSpace, as shown in Figure 8A. Recent emergence terms highlight the field's comprehensive advancement toward precision targeting, evidence‐based medicine, and renewed understanding of disease essence. As shown in Figure 8B, the co‐citation cluster analysis showed the most popular terms by hierarchical cluster labels, including 0# molecular pathway analysis, 1# comprehensive management and clinical guidelines, 2# pathogenesis and fibroblast research, 3# genetic susceptibility, 4# synergistic drug delivery, 5# photodynamic therapy, and 6# novel compound therapies. A timeline view of keyword cluster analysis is presented in Figure 8C. The results suggested that precision targeting and systemic management for keloids are currently emphasized in this field.

### 3.7 Analysis of Clinical Trial Progress

**FIGURE 9:**
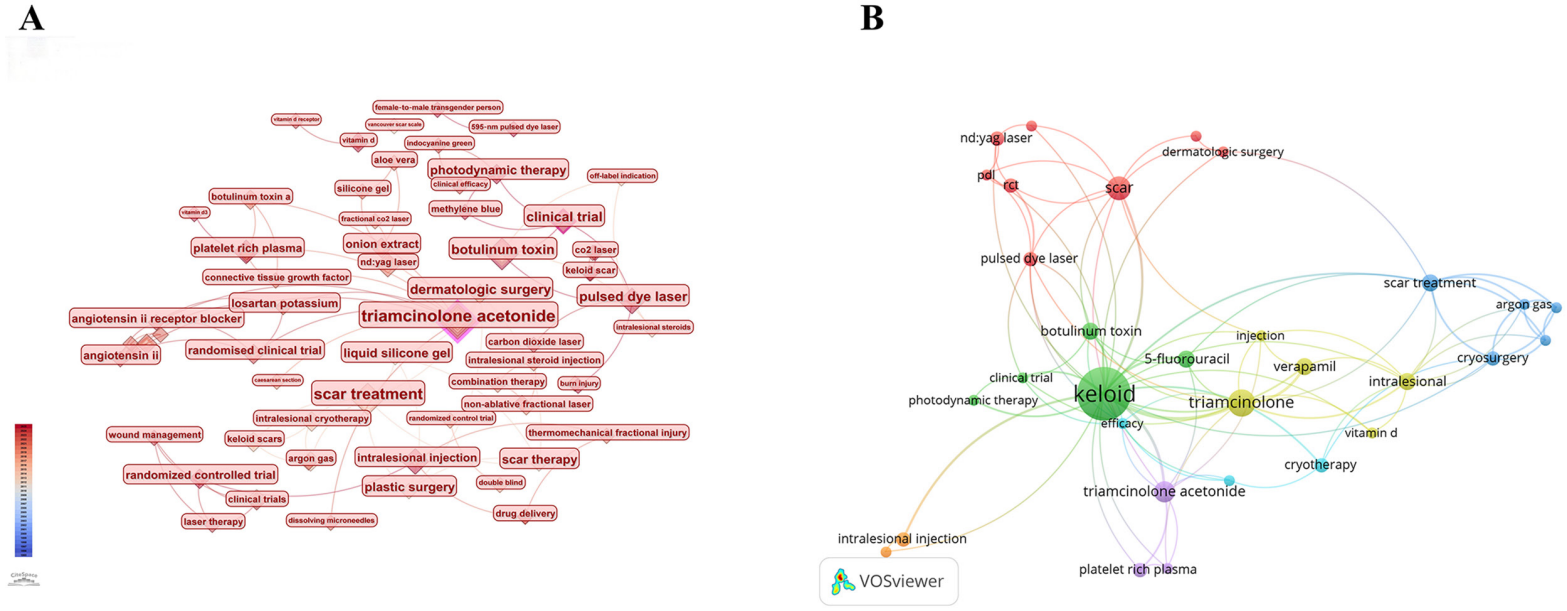
Keyword co‐occurrence map of clinical trial progress. (A) Visualization of the co‐occurrence by Citespace. (B) Visualization of the keyword overlay network.

PubMed, an internationally recognized biomedical literature database, contains a vast collection of high‐quality clinical research, particularly randomized controlled trials and systematic reviews. This study uses PubMed to address limitations in Web of Science data, systematically reviewing clinical advances in keloid treatment. It aims to reveal the latest clinical research trends and trial outcomes for healthcare practitioners. Figure 9 presents a co‐occurrence map of keywords in this field, highlighting current treatment hotspots in keloid management.

## 4 Discussion

We retrieved 1913 publications from the SCI‐E database of WOSCC between 1995 and 2025. Both the number of publications and citation frequency showed a steady upward trend, suggesting increasing research output, citation impact, and scholarly attention in the field of keloid treatment research. Overall, keloid remains an active field that warrants further academic investigation.

Collaborative network analyses enable the assessment of partnerships among countries, institutions, scholars, and journals. China was identified as one of the leading contributors in keloid treatment research. Several factors may help explain this pattern, including the potential clinical burden of keloids, demand for diverse treatment strategies, and growing academic interest in minimally invasive and integrative therapeutic approaches. The Chinese Academy of Medical Sciences and Peking Union Medical College also demonstrated high productivity in this field. This may be associated with their clinical resources, research infrastructure, and academic experience. Bayat, A. has published numerous highly cited papers on the biology of keloid fibroblasts and extracellular matrix regulation. Ogawa, R, whose research is renowned for rigorous clinical design and long‐term follow‐up data, has made significant contributions, particularly in stress therapy and radiotherapy regimens. Park, TH, is famous for his exceptional and innovative surgical techniques, particularly in the functional reconstruction and aesthetic restoration of complex keloids.

Analysis of source journals can help researchers identify the core journals in their research fields. Among the 10 most frequently cited journals in the field, *Dermatologic Surgery* published the most articles, followed by *Journal of Cosmetic Dermatology* and *Plastic and Reconstructive Surgery*. Articles published in *Plastic and Reconstructive Surgery* had the highest average citations per article, followed closely by *Journal of Investigative Dermatology*. These journals all focus on the intersection of dermatology and reconstructive plastic surgery, addressing core issues in keloid research, including abnormal repair processes after skin trauma and aesthetic and functional outcomes.

Overall, cooperation among countries, institutions, journals, and authors helps address scientific challenges and obstacles in the field by leveraging their respective strengths and characteristics to lay the groundwork for further research.

A keloid is a fibroproliferative disorder that can develop anywhere on the body [1]. Although keloids are classified as benign dermal growths, they exhibit biological features similar to those of malignant tumors, including genetic susceptibility, uncontrolled proliferation, invasiveness, treatment resistance, and recalcitrant recurrence [38, 39]. Keloids grow beyond the boundaries of the original wound, causing pain, pruritus, and contracture, which leads to a severe physical and psychological burden for patients [7].

Keloid formation is determined by multiple synergistically interacting genes, whose expression is regulated epigenetically and triggered by environmental stimuli [40]. Genes within keloid susceptibility loci, such as tumor necrosis factor‐α‐induced protein 6 (TNFAIP6) and acid sphingomyelinase (ASAH1), are highly expressed in various cancers and are involved in cellular invasion, migration, and anti‐apoptotic processes [41, 42]. At the genetic level, p53 dysfunction or mutations, along with imbalanced expression of pro‐apoptotic genes and anti‐apoptotic genes, disrupt normal regulation of programmed cell death [39]. Another mechanism of keloid pathogenesis involves epigenetics. Research indicates that imbalances in reversible epigenetic modifications may disrupt regular wound repair and lead to keloid formation, including DNA methylation, histone modifications, and non‐coding RNAs [39, 43]. These mechanisms collectively confer tumor‐like resistance to apoptosis on keloid cells, sustaining their abnormal survival and growth.

Keloid is regulated by multiple cell types, complex cytokine networks, and intertwined signaling pathways. Numerous growth factors are overexpressed and secreted in keloids, including transforming growth factor‐β (TGF‐β), insulin‐like growth factor I (IGF‐I), platelet‐derived growth factor (PDGF), and epidermal growth factor (EGF) [44, 45, 46]. Keratinocytes, fibroblasts, endothelial cells, and immune cells (e.g., mast cells, macrophages, and lymphocytes) collectively contribute to keloid formation by regulating multiple signaling pathways [47]. For example, mast cells promote sustained fibroblast activation through pathways including PI3K/Akt/mTOR and TGF‐β1/Smad. Macrophages further encourage fibroblast proliferation and extracellular matrix deposition by secreting cytokines such as TGF‐β and PDGF. Additionally, alterations in the expression levels of matrix metalloproteinases (MMPs) and their inhibitors may dysregulate collagen synthesis and degradation [47]. Collectively, these alterations result in excessive collagen deposition in keloids, leading to their invasive growth pattern [48].

Moreover, keloids sustain their continuous growth through abnormal proliferation of the vascular network, epithelial‐mesenchymal transition (EMT), hypoxia, and inflammation [47, 49, 50]. Pro‐angiogenic factors are significantly upregulated, accompanied by the recruitment of peripheral blood‐derived active endothelial progenitor cells to the lesion site, resulting in the rich vascular architecture characteristic of keloids [51]. Additionally, keloids become invasive through EMT, characterized by decreased E‐cadherin and increased vimentin expression, which enhances cellular migration capacity [50]. Additionally, keloid formation involves the synergistic interaction between metabolic reprogramming and chronic inflammation [49]. Fibroblasts prefer aerobic glycolysis, exhibiting high oxygen consumption, elevated lactate levels, and increased ATP production to fuel cellular proliferation and collagen synthesis. Concurrently, chronic inflammation persists within the lesion, characterized by elevated pro‐inflammatory factors and reduced anti‐inflammatory factors [47]. In summary, these factors collectively promote abnormal keloid proliferation.

In short, keloids exhibit biological behavior more akin to a benign yet locally invasive skin tumor. Consequently, proactive intervention and comprehensive management strategies are urgently needed to reduce recurrence rates and improve patient outcomes.

The emergence of keywords such as “efficacy,” “safety,” and “double‐blind” signifies the transformation of keloid treatment research into a modern clinical discipline grounded in evidence‐based practices.

First, research methodologies are advancing toward an era of advanced evidence‐based medicine. Research designs are increasingly adopting multicenter, randomized, double‐blind, and controlled approaches [52]. Given the complexity and recurrence tendency of keloids, new therapies (such as targeted drugs and combination therapies) are being compared head‐to‐head with current standard treatments (such as corticosteroid injections) [53, 54].

Meanwhile, evaluation metrics tend to be multidimensional, quantifiable indicators [55]. For instance, high‐resolution imaging techniques and three‐dimensional imaging systems can quantify keloid size and volume [56, 57]. Quantifying pathological and molecular alterations is equally crucial, for example, using immunohistochemical staining to measure fibroblast density, collagen type ratios, and expression levels of key cytokines [58]. Furthermore, the integrated use of assessment scales is vital. Combining tools such as the Vancouver Scar Scale (VSS), Visual Analog Scale (VAS), and Dermatology Life Quality Index (DLQI) enables the integration of subjective patient perceptions with objective clinical findings, providing a comprehensive reflection of the treatment's impact on both physical and psychological well‐being [59, 60].

Ultimately, the goal of clinical practice is shifting toward a patient‐centered, personalized medicine model. By identifying biomarkers that predict disease risk, prognosis, and treatment response, this approach paves the way for personalized medicine based on precise classification and targeted therapies [61, 62]. Treatment is not limited to improving scar appearance; it prioritizes achieving tangible relief from pain and itching, restoring social functioning, and maintaining psychological well‐being [59, 60, 63].

In summary, the treatment of keloids should strike a balance between evidence‐based standardized care and personalized approaches, ultimately aiming to enhance patients' overall health and quality of life.

Conventional treatments for keloids include physical therapy, intralesional injections, invasive interventions, and biological therapy [64, 65, 66, 67, 68, 69, 70, 71].

Silicone dressing is a non‐invasive treatment option for keloids that works by sealing the skin and providing moisturization [72]. It works by hydrating the stratum corneum and regulating cellular signaling between fibroblasts and keratinocytes [73]. This method progressively improves scar color, size, erythema, and flexibility while alleviating pain and itching, offering both safety and efficacy [74]. Additionally, pressure therapy is often combined with other treatments to suppress keloid proliferation and promote scar maturation and softening through sustained, uniform mechanical pressure. Laser therapy is a commonly used method that selectively targets abnormal collagen tissue with high‐energy beams to remodel its structure while minimizing damage to surrounding healthy tissue [75]. Standard techniques include ablative carbon dioxide lasers, pulsed dye lasers (PDLs), and fractional lasers [76, 77]. These therapies effectively reduce scar size and thickness and alleviate erythema, but may cause side effects such as pain, hyperpigmentation, and infection. Cryotherapy directly damages cells within keloids through rapid freezing, inducing microcirculatory injury that leads to ischemic necrosis, thereby further disrupting the skin lesion [78]. In recent years, photodynamic therapy (PDT) based on 5‐aminolevulinic acid (5‐ALA) has garnered significant attention. As a precursor in heme biosynthesis, 5‐ALA permeates mitochondria and is enzymatically converted into porphyrin IX. This compound reacts with laser light to generate reactive oxygen species (ROS), ultimately inducing cell death [79].

Intralesional injection delivers therapeutic agents directly into the skin lesion, targeting pathological mechanisms to reduce keloid volume, alleviate symptoms, and lower the risk of recurrence [80]. Corticosteroids are commonly used medications that prevent and treat keloids through anti‐inflammatory, immunomodulatory, antifibroblastic, antiangiogenic, and Extracellular Matrix (ECM) remodeling effects [81]. Patients must be closely monitored for potential side effects, including skin atrophy, hypopigmentation, and telangiectasia [64]. Certain chemotherapy drugs, such as bleomycin, 5‐fluorouracil, and mitomycin C, have also been shown to effectively treat and prevent keloids by targeting fibroblasts in scar tissue, inducing apoptosis, or regulating protein synthesis [82, 83, 84].

Surgery and radiotherapy are often combined to treat keloids [85]. Small keloids may be excised entirely, while larger or multiple keloids may be treated with partial or core excision [86]. However, radical or total excision of keloids may stimulate collagen synthesis, increasing the risk of recurrence and potentially resulting in keloids larger than the original lesions. Radiotherapy is a standard adjunctive treatment following keloid excision, aiming to reduce recurrence rates by inhibiting angiogenesis and fibroblast proliferation [87].

Platelet‐rich plasma therapy is a regenerative treatment based on autologous blood extraction [88]. This method involves collecting the patient's own blood, centrifuging it to concentrate the platelets, and injecting the platelets into the scar tissue. Upon activation, platelets release multiple growth factors (such as platelet‐derived growth factor, transforming growth factor‐β, and vascular endothelial growth factor). These factors modulate local inflammatory responses, suppress excessive fibrosis, and promote the orderly arrangement of collagen fibers and tissue remodeling [89]. Clinical studies demonstrate that this therapy improves scar hardness, color, and elasticity. It is often combined with corticosteroid injections or surgical excision to enhance efficacy and reduce recurrence rates [90].

Although keloids can be treated using multiple approaches, no single method is completely effective or satisfactory [7]. In our research, the keywords “delivery” and “injection” have experienced explosive growth, highlighting a shift in research focus toward localized precision drug delivery [91, 92]. This approach aims to enhance local drug concentration, minimize systemic side effects, and achieve precise therapeutic targeting.

In fact, the treatment of keloids has long faced significant challenges in drug delivery. First, the dense collagen matrix within the lesion severely impedes drug penetration and diffusion [92]. Second, traditional intra‐lesional injection methods suffer from uneven drug distribution, failing to precisely distinguish and target the fibroblast cells within the lesion [93]. Furthermore, drugs injected into the tissue are often rapidly cleared by local blood circulation, resulting in short drug residence times and requiring patients to undergo frequent treatments. Finally, drugs may leak from the injection site into the systemic circulation, triggering unnecessary systemic side effects [64].

To address these challenges, novel drug delivery technologies are rapidly advancing across multiple dimensions. Intralesional injection techniques themselves are being optimized. For instance, radionuclide‐labeled intralesional injections can ensure precise drug placement within the keloid core and significantly enhance efficacy [57, 80]. Improved multi‐point, superficial injection techniques further aid in achieving more uniform drug distribution within tissue. Concurrently, microneedle technology offers a minimally invasive alternative [94]. This approach not only enables patient self‐administration to improve compliance but also facilitates sustained or on‐demand drug delivery [95]. Notably, dissolvable microneedles degrade after releasing their payload [96].

Additionally, sustained‐release carriers are specifically designed to prolong drug action, enabling continuous release at the target site for weeks after injection and significantly reducing dosing frequency [26, 97]. Local injection of hydrogels offers an attractive therapeutic approach because it can be administered without intravenous access, enabling sustained drug release and high‐dose delivery at the lesion site [98]. More advanced targeted delivery systems achieve precision by attaching antibodies, peptides, or aptamers to nanocarriers that specifically recognize markers overexpressed on the surface of scar fibroblasts [99, 100]. Response‐triggered release carriers can be engineered to release drugs exclusively within specific microenvironments (e.g., low pH, specific enzymes, or oxidative stress conditions), thereby further enhancing therapeutic specificity and safety [101, 102].

Additionally, technologies that enhance physical assisted delivery provide powerful tools for overcoming tissue barriers [103]. Laser‐assisted drug delivery technology uses fractional lasers to create microscopic treatment zones on the skin, enabling topical medications to penetrate deeply for more efficient drug delivery [63]. Ultrasound or iontophoresis technologies directly promote transdermal drug penetration through physical energy [103]. For post‐surgical wound management, 3D‐printed bioscaffolds serve as implantable devices that combine sustained‐release drug delivery with guidance for tissue regeneration [104].

Modern drug delivery technologies enhance local drug concentration and retention time while reducing systemic exposure risks, enabling precise targeting of diseased cells. The future trend will be the deep integration of targeted drugs and delivery systems.

This study possesses multiple advantages. First, by employing various bibliometric tools such as VOSviewer, Scimago Graphic, and CiteSpace, it visualizes keyword co‐occurrence, collaborative networks, and citation bursts. Second, the study introduces a clinical progress analysis to systematically map treatment trends. It not only documents the evolving clinical roles of traditional therapies such as surgery, radiotherapy, and drug injections but also reveals the emergence of novel treatment concepts based on signaling pathway regulation, metabolic reprogramming, and microenvironment remodeling. This provides a systematic overview of the developmental pathways and future directions for clinical practice in this field.

However, this study still has limitations. First, this study relied solely on data from the WoSCC and PubMed, potentially excluding relevant studies from other databases, which could limit the analysis's comprehensiveness. In addition, the database‐specific coverage and indexing policies of WoSCC and PubMed may have introduced indexing bias, particularly by underrepresenting studies published in regional journals or emerging research areas. Second, this study included only English‐language publications, making it susceptible to language bias. Third, this study mainly focused on quantitative bibliometric indicators, without directly evaluating the quality of individual studies. Citation‐based indicators may also introduce citation bias, as highly cited publications do not necessarily indicate higher quality, while recently published or less visible studies may be underestimated.

## 5 Conclusion

In conclusion, this study elucidates the knowledge structure and developmental trends of keloid treatment through bibliometric analysis. The sustained growth in the number of publications indicates increasing research activity in this field. Based on the identified research trends, several emerging therapeutic directions appear to be gaining attention. Given the “tumor‐like” characteristics of keloids, studies targeting abnormally activated signaling pathways, precision inhibitors, and metabolic reprogramming have emerged as potential areas for future investigation. In addition, increasing attention has been directed toward the pro‐fibrotic local microenvironment, including hypoxia, chronic inflammation, and immunosuppressive states. Personalized treatment strategies based on molecular subtyping or genetic characteristics, particularly in combination therapy selection, also represent an evolving research trend that may contribute to the transition from symptomatic management toward mechanism‐oriented intervention.

## Author Contributions

Chengxin Li and Rui Wang raised the conception of the study and designed the study. Yilin Wang, Jintong Wu, and Zijie Tang conducted the CiteSpace and VOSviewer analysis. Yilin Wang and Jintong Wu screened articles and wrote the original manuscript. Chengxin Li and Rui Wang revised the manuscript and edited it critically. All authors contributed to the article and approved the submitted version. Yilin Wang and Jintong Wu contributed equally to this study.

## Funding

This study was supported by the National Natural Science Foundation of China (Nos. 82504266, 82273530, 81972936‐JT).

## Ethics Statement

The authors have nothing to report.

## Conflicts of Interest

The authors declare no conflicts of interest.

## Supporting information


**Appendix S1:** jocd71054‐sup‐0001‐AppendixS1.docx.

## Data Availability

All datasets presented in the study are included in the article. Further inquiries can be directed to the corresponding authors.

## References

[1] Y. Liu , X. Chen , K. S. Fischer , S. Fu , L. Yuan , and X. Hu , “Keloids Revisited: Current Concepts in Treatment and Differential Diagnosis,” Cancer Letters 625 (2025): 217802, 10.1016/j.canlet.2025.217802. 40374155

[2] G. H. M. Stanley , E. R. Pitt , D. Lim , and J. Pleat , “Prevalence, Exposure and the Public Knowledge of Keloids on Four Continents,” Journal of Plastic, Reconstructive & Aesthetic Surgery 77 (2023): 359–370, 10.1016/j.bjps.2022.11.017. 36621239

[3] A. Bayat , D. A. McGrouther , and M. W. Ferguson , “Skin Scarring,” BMJ (Clinical Research ed.) 326, no. 7380 (2003): 88–92, 10.1136/bmj.326.7380.88. PMC1125033 12521975

[4] Y. Y. Lu , H. P. Tu , C. H. Wu , et al., “Risk of Cancer Development in Patients With Keloids,” Scientific Reports 11, no. 1 (2021): 9390, 10.1038/s41598-021-88789-1. 33931723 PMC8087779

[5] D. D. Balci , T. Inandi , C. A. Dogramaci , and E. Celik , “DLQI Scores in Patients With Keloids and Hypertrophic Scars: A Prospective Case Control Study,” Journal of the German Society of Dermatology 7, no. 8 (2009): 688–692, 10.1111/j.1610-0387.2009.07034.x. 19243478

[6] T. Murakami and S. Shigeki , “Pharmacotherapy for Keloids and Hypertrophic Scars,” International Journal of Molecular Sciences 25, no. 9 (2024): 4674, 10.3390/ijms25094674. 38731893 PMC11083137

[7] H. J. Kim and Y. H. Kim , “Comprehensive Insights Into Keloid Pathogenesis and Advanced Therapeutic Strategies,” International Journal of Molecular Sciences 25, no. 16 (2024): 8776, 10.3390/ijms25168776. 39201463 PMC11354446

[8] S. Schoepe , H. Schäcke , E. May , and K. Asadullah , “Glucocorticoid Therapy‐Induced Skin Atrophy,” Experimental Dermatology 15, no. 6 (2006): 406–420, 10.1111/j.0906-6705.2006.00435.x. 16689857

[9] X. Dong , T. Wang , C. Gao , Y. Cui , and L. Li , “Multi‐Target Pharmacological Effects of Asiatic Acid: Advances in Structural Modification and Novel Drug Delivery Systems,” Molecules 30, no. 18 (2025): 3688, 10.3390/molecules30183688. 41011581 PMC12473031

[10] Z. M. Zhuang , Y. Wang , Z. X. Feng , et al., “Targeting Diverse Wounds and Scars: Recent Innovative Bio‐Design of Microneedle Patch for Comprehensive Management,” Small (Weinheim an der Bergstrasse, Germany) 20, no. 18 (2024): e2306565, 10.1002/smll.202306565. 38037685

[11] J. P. Andrews , J. Marttala , E. Macarak , J. Rosenbloom , and J. Uitto , “Keloids: The Paradigm of Skin Fibrosis—Pathomechanisms and Treatment,” Matrix Biology 51 (2016): 37–46, 10.1016/j.matbio.2016.01.013. 26844756 PMC4842154

[12] M. Aria and C. Cuccurullo , “Bibliometrix: An R‐Tool for Comprehensive Science Mapping Analysis,” Journal of Informetrics 11, no. 4 (2017): 959–975.

[13] G. Cabanac , I. Frommholz , and P. Mayr , “Scholarly Literature Mining With Information Retrieval and Natural Language Processing: Preface,” Scientometrics 125, no. 3 (2020): 2835–2840, 10.1007/s11192-020-03763-4. 33223580 PMC7670972

[14] N. J. van Eck and L. Waltman , “Software Survey: VOSviewer, a Computer Program for Bibliometric Mapping,” Scientometrics 84, no. 2 (2009): 523–538, 10.1007/s11192-009-0146-3. 20585380 PMC2883932

[15] Y. Hu , Z. Yu , X. Cheng , Y. Luo , and C. Wen , “A Bibliometric Analysis and Visualization of Medical Data Mining Research,” Medicine 99, no. 22 (2020): e20338, 10.1097/md.0000000000020338. 32481411 PMC7748217

[16] I. D. Cooper , “Bibliometrics Basics,” Journal of the Medical Library Association 103, no. 4 (2015): 217–218, 10.3163/1536-5050.103.4.013. 26512226 PMC4613387

[17] J. E. Hirsch , “An Index to Quantify an Individual's Scientific Research Output,” National Academy of Sciences of the United States of America 102, no. 46 (2005): 16569–16572, 10.1073/pnas.0507655102. PMC1283832 16275915

[18] R. Bagabir , R. J. Byers , I. H. Chaudhry , W. Müller , R. Paus , and A. Bayat , “Site‐Specific Immunophenotyping of Keloid Disease Demonstrates Immune Upregulation and the Presence of Lymphoid Aggregates,” British Journal of Dermatology 167, no. 5 (2012): 1053–1066, 10.1111/j.1365-2133.2012.11190.x. 23106354

[19] R. Ogawa , “The Most Current Algorithms for the Treatment and Prevention of Hypertrophic Scars and Keloids: A 2020 Update of the Algorithms Published 10 Years Ago,” Plastic and Reconstructive Surgery 149, no. 1 (2022): 79e–94e, 10.1097/prs.0000000000008667. PMC8687618 34813576

[20] T. H. Park , S. W. Seo , J. K. Kim , and C. H. Chang , “Outcomes of Surgical Excision With Pressure Therapy Using Magnets and Identification of Risk Factors for Recurrent Keloids,” Plastic and Reconstructive Surgery 128, no. 2 (2011): 431–439, 10.1097/PRS.0b013e31821e7006. 21788835

[21] Z. Wang , C. Feng , K. Song , Z. Qi , W. Huang , and Y. Wang , “lncRNA‐H19/miR‐29a Axis Affected the Viability and Apoptosis of Keloid Fibroblasts Through Acting Upon COL1A1 Signaling,” Journal of Cellular Biochemistry 121, no. 11 (2020): 4364–4376, 10.1002/jcb.29649. 31930556

[22] C. P. Lim , T. T. Phan , I. J. Lim , and X. Cao , “Stat3 Contributes to Keloid Pathogenesis via Promoting Collagen Production, Cell Proliferation and Migration,” Oncogene 25, no. 39 (2006): 5416–5425, 10.1038/sj.onc.1209531. 16619044

[23] G. C. Limandjaja , F. B. Niessen , R. J. Scheper , and S. Gibbs , “The Keloid Disorder: Heterogeneity, Histopathology, Mechanisms and Models,” Frontiers in Cell and Developmental Biology 8 (2020): 360, 10.3389/fcell.2020.00360. 32528951 PMC7264387

[24] Y. Hao , X. Dong , M. Zhang , H. Liu , L. Zhu , and Y. Wang , “Effects of Hyperbaric Oxygen Therapy on the Expression Levels of the Inflammatory Factors Interleukin‐12p40, Macrophage Inflammatory Protein‐1β, Platelet‐Derived Growth Factor‐BB, and Interleukin‐1 Receptor Antagonist in Keloids,” Medicine (Baltimore) 99, no. 16 (2020): e19857, 10.1097/md.0000000000019857. 32312010 PMC7220187

[25] H. Liu , X. Chi , N. Yang , et al., “Joint Effect of RRP9 and DDX21 on Development of Colorectal Cancer and Keloid,” Aging 15, no. 24 (2023): 14703–14719, 10.18632/aging.205240. 37988222 PMC10781455

[26] M. Wang , L. Chen , W. Huang , et al., “Improving the Anti‐Keloid Outcomes Through Liposomes Loading Paclitaxel‐Cholesterol Complexes,” International Journal of Nanomedicine 14 (2019): 1385–1400, 10.2147/ijn.S195375. 30863067 PMC6390862

[27] J. H. Park , J. Y. Chun , and J. H. Lee , “Laser‐Assisted Topical Corticosteroid Delivery for the Treatment of Keloids,” Lasers in Medical Science 32, no. 3 (2017): 601–608, 10.1007/s10103-017-2154-5. 28124198

[28] G. G. Gauglitz , H. C. Korting , T. Pavicic , T. Ruzicka , and M. G. Jeschke , “Hypertrophic Scarring and Keloids: Pathomechanisms and Current and Emerging Treatment Strategies,” Molecular Medicine (Cambridge, Mass.) 17, no. 1–2 (2011): 113–125, 10.2119/molmed.2009.00153. 20927486 PMC3022978

[29] M. Xue and C. J. Jackson , “Extracellular Matrix Reorganization During Wound Healing and Its Impact on Abnormal Scarring,” Advances in Wound Care 4, no. 3 (2015): 119–136, 10.1089/wound.2013.0485. 25785236 PMC4352699

[30] T. A. Mustoe , R. D. Cooter , M. H. Gold , et al., “International Clinical Recommendations on Scar Management,” Plastic and Reconstructive Surgery 110, no. 2 (2002): 560–571, 10.1097/00006534-200208000-00031. 12142678

[31] R. Ogawa , “Keloid and Hypertrophic Scars Are the Result of Chronic Inflammation in the Reticular Dermis,” International Journal of Molecular Sciences 18, no. 3 (2017): 606, 10.3390/ijms18030606. 28287424 PMC5372622

[32] F. B. Niessen , P. H. Spauwen , J. Schalkwijk , and M. Kon , “On the Nature of Hypertrophic Scars and Keloids: A Review,” Plastic and Reconstructive Surgery 104, no. 5 (1999): 1435–1458, 10.1097/00006534-199910000-00031. 10513931

[33] B. Berman , A. Maderal , and B. Raphael , “Keloids and Hypertrophic Scars: Pathophysiology, Classification, and Treatment,” Dermatologic Surgery 43, no. Suppl 1 (2017): S3–S18, 10.1097/dss.0000000000000819. 27347634

[34] D. Wolfram , A. Tzankov , P. Pülzl , and H. Piza‐Katzer , “Hypertrophic Scars and Keloids—A Review of Their Pathophysiology, Risk Factors, and Therapeutic Management,” Dermatologic Surgery 35, no. 2 (2009): 171–181, 10.1111/j.1524-4725.2008.34406.x. 19215252

[35] A. Al‐Attar , S. Mess , J. M. Thomassen , C. L. Kauffman , and S. P. Davison , “Keloid Pathogenesis and Treatment,” Plastic and Reconstructive Surgery 117, no. 1 (2006): 286–300, 10.1097/01.prs.0000195073.73580.46. 16404281

[36] O. Bock , G. Schmid‐Ott , P. Malewski , and U. Mrowietz , “Quality of Life of Patients With Keloid and Hypertrophic Scarring,” Archives of Dermatological Research 297, no. 10 (2006): 433–438, 10.1007/s00403-006-0651-7. 16528552

[37] H. J. Lee and Y. J. Jang , “Recent Understandings of Biology, Prophylaxis and Treatment Strategies for Hypertrophic Scars and Keloids,” International Journal of Molecular Sciences 19, no. 3 (2018): 711, 10.3390/ijms19030711. 29498630 PMC5877572

[38] M. Abu‐Sultanah , Z. Zhou , C. Jiang , et al., “TGFβ‐Dependent Signaling Drives Tumor Growth and Aberrant Extracellular Matrix Dynamics in NF1‐Associated Plexiform Neurofibroma,” Science Advances 11, no. 25 (2025): eadu0772, 10.1126/sciadv.adu0772. 40540562 PMC12180511

[39] D. T. Nyika , N. P. Khumalo , and A. Bayat , “Genetics and Epigenetics of Keloids,” Advances in Wound Care 11, no. 4 (2022): 192–201, 10.1089/wound.2021.0094. 34498914

[40] H. Wang , Z. Zhou , Y. Liu , et al., “Identification and Validation of HOXD3 and UNC5C as Molecular Signatures in Keloid Based on Weighted Gene Co‐Expression Network Analysis,” Genomics 114, no. 4 (2022): 110403, 10.1016/j.ygeno.2022.110403. 35709926

[41] C. Zhong , K. Shi , P. Li , et al., “Single‐Cell Sequencing Analysis and Bulk‐Seq Identify IGFBP6 and TNFAIP6 as Novel Differential Diagnosis Markers for Postburn Pathological Scarring,” Burns 50, no. 9 (2024): 107255, 10.1016/j.burns.2024.08.021. 39317554

[42] R. L. P. Santos‐Cortez , Y. Hu , F. Sun , et al., “Identification of ASAH1 as a Susceptibility Gene for Familial Keloids,” European Journal of Human Genetics 25, no. 10 (2017): 1155–1161, 10.1038/ejhg.2017.121. 28905881 PMC5602022

[43] S. Liu , H. Yang , J. Song , Y. Zhang , A. T. H. Abualhssain , and B. Yang , “Keloid: Genetic Susceptibility and Contributions of Genetics and Epigenetics to Its Pathogenesis,” Experimental Dermatology 31, no. 11 (2022): 1665–1675, 10.1111/exd.14671. 36052657

[44] Y. K. Hong , Y. C. Lin , T. L. Cheng , et al., “TEM1/Endosialin/CD248 Promotes Pathologic Scarring and TGF‐β Activity Through Its Receptor Stability in Dermal Fibroblasts,” Journal of Biomedical Science 31, no. 1 (2024): 12, 10.1186/s12929-024-01001-0. 38254097 PMC10804696

[45] C. Guo , L. Liang , J. Zheng , et al., “UCHL1 Aggravates Skin Fibrosis Through an IGF‐1‐Induced Akt/mTOR/HIF‐1α Pathway in Keloid,” FASEB Journal 37, no. 7 (2023): e23015, 10.1096/fj.202300153RR. 37256780

[46] B. Y. Zhou , W. B. Wang , X. L. Wu , et al., “Nintedanib Inhibits Keloid Fibroblast Functions by Blocking the Phosphorylation of Multiple Kinases and Enhancing Receptor Internalization,” Acta Pharmacologica Sinica 41, no. 9 (2020): 1234–1245, 10.1038/s41401-020-0381-y. 32327724 PMC7608201

[47] Y. Li , M. Li , C. Qu , et al., “The Polygenic Map of Keloid Fibroblasts Reveals Fibrosis‐Associated Gene Alterations in Inflammation and Immune Responses,” Frontiers in Immunology 12 (2021): 810290, 10.3389/fimmu.2021.810290. 35082796 PMC8785650

[48] S. Vimalraj , “A Concise Review of VEGF, PDGF, FGF, Notch, Angiopoietin, and HGF Signalling in Tumor Angiogenesis With a Focus on Alternative Approaches and Future Directions,” International Journal of Biological Macromolecules 221 (2022): 1428–1438, 10.1016/j.ijbiomac.2022.09.129. 36122781

[49] Q. Wang , P. Wang , Z. Qin , et al., “Altered Glucose Metabolism and Cell Function in Keloid Fibroblasts Under Hypoxia,” Redox Biology 38 (2021): 101815, 10.1016/j.redox.2020.101815. 33278780 PMC7718484

[50] Z. K. Qiu , E. Yang , N. Z. Yu , et al., “The Biomarkers Associated With Epithelial‐Mesenchymal Transition in Human Keloids,” Burns 50, no. 2 (2024): 474–487, 10.1016/j.burns.2023.09.009. 37980270

[51] X. Liu , W. Chen , Q. Zeng , et al., “Single‐Cell RNA‐Sequencing Reveals Lineage‐Specific Regulatory Changes of Fibroblasts and Vascular Endothelial Cells in Keloids,” Journal of Investigative Dermatology 142, no. 1 (2022): 124–135.e11, 10.1016/j.jid.2021.06.010. 34242659

[52] M. S. Nestor , B. Berman , D. L. Fischer , et al., “A Randomized, Double‐Blind, Active‐ and Placebo‐Controlled Trial Evaluating a Novel Topical Treatment for Keloid Scars,” Journal of Drugs in Dermatology 20, no. 9 (2021): 964–968, 10.36849/jdd.6197. 34491021

[53] E. S. Hewedy , B. E. I. Sabaa , W. S. Mohamed , and D. S. Hegab , “Combined Intralesional Triamcinolone Acetonide and Platelet Rich Plasma Versus Intralesional Triamcinolone Acetonide Alone in Treatment of Keloids,” Journal of Dermatological Treatment 33, no. 1 (2022): 150–156, 10.1080/09546634.2020.1730742. 32063079

[54] W. Disphanurat , N. Sivapornpan , B. Srisantithum , and J. Leelawattanachai , “Efficacy of a Triamcinolone Acetonide‐Loaded Dissolving Microneedle Patch for the Treatment of Hypertrophic Scars and Keloids: A Randomized, Double‐Blinded, Placebo‐Controlled Split‐Scar Study,” Archives of Dermatological Research 315, no. 4 (2023): 989–997, 10.1007/s00403-022-02473-6. 36383222

[55] J. Chambert , T. Lihoreau , S. Joly , et al., “Multimodal Investigation of a Keloid Scar by Combining Mechanical Tests In Vivo With Diverse Imaging Techniques,” Journal of the Mechanical Behavior of Biomedical Materials 99 (2019): 206–215, 10.1016/j.jmbbm.2019.07.025. 31374516

[56] L. Nguyen , T. Dohi , H. Watanabe‐Takano , S. Fukuhara , and R. Ogawa , “Comprehensive Analysis of Keloid Vasculature by Tissue Clearing and 3D Imaging,” Wound Repair and Regeneration 33, no. 2 (2025): e70015, 10.1111/wrr.70015. 40143403 PMC11947297

[57] L. Zhou , Q. Zhou , C. Zheng , Z. Wang , and M. Rao , “Multimodal Ultrasound Assessment for Monitoring Keloid Severity and Treatment Response,” Scientific Reports 15, no. 1 (2025): 8568, 10.1038/s41598-025-91111-y. 40074795 PMC11903770

[58] J. Shim , S. J. Oh , E. Yeo , et al., “Integrated Analysis of Single‐Cell and Spatial Transcriptomics in Keloids: Highlights on Fibrovascular Interactions in Keloid Pathogenesis,” Journal of Investigative Dermatology 142, no. 8 (2022): 2128–2139.e11, 10.1016/j.jid.2022.01.017. 35123990

[59] R. M. Bernabe , P. Madrigal , D. Choe , C. Pham , H. A. Yenikomshian , and J. Gillenwater , “Assessing Scar Outcomes Using Objective Scar Measurement Tools: An Adjunct to Validated Scar Evaluation Scales,” Plastic and Reconstructive Surgery 154, no. 5 (2024): 885e–890e, 10.1097/prs.0000000000011424. 38546618

[60] S. Sitaniya , D. Subramani , A. Jadhav , Y. K. Sharma , M. S. Deora , and A. Gupta , “Quality‐Of‐Life of People With Keloids and Its Correlation With Clinical Severity and Demographic Profiles,” Wound Repair and Regeneration 30, no. 3 (2022): 409–416, 10.1111/wrr.13015. 35388938

[61] W. Zhang , F. Wang , Y. Zhang , et al., “New Insights Into Keloid Pathogenesis: Biomarker Potential for CDK7 and DDB2,” Frontiers in Cell and Developmental Biology 13 (2025): 1718189, 10.3389/fcell.2025.1718189. 41347148 PMC12672422

[62] T. Mishra and S. Wairkar , “Pathogenesis, Attenuation, and Treatment Strategies for Keloid Management,” Tissue & Cell 94 (2025): 102800, 10.1016/j.tice.2025.102800. 39999656

[63] J. G. Labadie , S. A. Ibrahim , B. Worley , et al., “Evidence‐Based Clinical Practice Guidelines for Laser‐Assisted Drug Delivery,” JAMA Dermatology 158, no. 10 (2022): 1193–1201, 10.1001/jamadermatol.2022.3234. 35976634

[64] M. Sheng , Y. Chen , H. Li , Y. Zhang , and Z. Zhang , “The Application of Corticosteroids for Pathological Scar Prevention and Treatment: Current Review and Update,” Burns & Trauma 11 (2023): tkad009, 10.1093/burnst/tkad009. 36950503 PMC10025010

[65] R. Leszczynski , C. A. da Silva , A. Pinto , U. Kuczynski , and E. M. da Silva , “Laser Therapy for Treating Hypertrophic and Keloid Scars,” Cochrane Database of Systematic Reviews 9, no. 9 (2022): Cd011642, 10.1002/14651858.CD011642.pub2. 36161591 PMC9511989

[66] L. O'Brien and D. J. Jones , “Silicone Gel Sheeting for Preventing and Treating Hypertrophic and Keloid Scars,” Cochrane Database of Systematic Reviews 2013, no. 9 (2013): Cd003826, 10.1002/14651858.CD003826.pub3. 24030657 PMC7156908

[67] A. Sadeghinia and S. Sadeghinia , “Comparison of the Efficacy of Intralesional Triamcinolone Acetonide and 5‐Fluorouracil Tattooing for the Treatment of Keloids,” Dermatologic Surgery 38, no. 1 (2012): 104–109, 10.1111/j.1524-4725.2011.02137.x. 22093096

[68] P. L. Danielsen , S. M. Rea , F. M. Wood , et al., “Verapamil Is Less Effective Than Triamcinolone for Prevention of Keloid Scar Recurrence After Excision in a Randomized Controlled Trial,” Acta Dermato‐Venereologica 96, no. 6 (2016): 774–778, 10.2340/00015555-2384. 26911400

[69] A. A. Tawfik and R. A. Ali , “Evaluation of Botulinum Toxin Type A for Treating Post Burn Hypertrophic Scars and Keloid in Children: An Intra‐Patient Randomized Controlled Study,” Journal of Cosmetic Dermatology 22, no. 4 (2023): 1256–1260, 10.1111/jocd.15634. 36718819

[70] S. A. Ismail , N. H. K. Mohammed , M. Sotohy , and D. A. E. Abou‐Taleb , “Botulinum Toxin Type A Versus 5‐Fluorouracil in Treatment of Keloid,” Archives of Dermatological Research 313, no. 7 (2021): 549–556, 10.1007/s00403-020-02132-8. 32892246

[71] S. Kuribayashi , T. Miyashita , Y. Ozawa , et al., “Post‐Keloidectomy Irradiation Using High‐Dose‐Rate Superficial Brachytherapy,” Journal of Radiation Research 52, no. 3 (2011): 365–368, 10.1269/jrr.10159. 21490411

[72] M. Sandhofer and P. Schauer , “The Safety, Efficacy, and Tolerability of a Novel Silicone Gel Dressing Following Dermatological Surgery,” Skinmed 10, no. 6 (2012): S1–S7. 23346665

[73] J. S. Kim , J. P. Hong , J. W. Choi , D. K. Seo , E. S. Lee , and H. S. Lee , “The Efficacy of a Silicone Sheet in Postoperative Scar Management,” Advances in Skin & Wound Care 29, no. 9 (2016): 414–420, 10.1097/01.ASW.0000488665.03896.3d. 27538109

[74] F. Tian , Q. Jiang , J. Chen , and Z. Liu , “Silicone Gel Sheeting for Treating Keloid Scars,” Cochrane Database of Systematic Reviews 1, no. 1 (2023): Cd013878, 10.1002/14651858.CD013878.pub2. 36594476 PMC9808890

[75] M. P. Brewin , S. Docherty , V. Heaslip , et al., “Early Laser for Burn Scars (ELABS)—Randomised Controlled Trial of Pulsed Dye Laser Treatment and Standard Care Versus Standard Care Alone for the Treatment of Hypertrophic Burn Scars,” Burns 51, no. 5 (2025): 107500, 10.1016/j.burns.2025.107500. 40319828

[76] M. K. Kivi , A. Jafarzadeh , F. S. Hosseini‐Baharanchi , S. Salehi , and A. Goodarzi , “The Efficacy, Satisfaction, and Safety of Carbon Dioxide (CO_2_) Fractional Laser in Combination With Pulsed Dye Laser (PDL) Versus Each One Alone in the Treatment of Hypertrophic Burn Scars: A Single‐Blinded Randomized Controlled Trial,” Lasers in Medical Science 39, no. 1 (2024): 69, 10.1007/s10103-024-03976-6. 38376542

[77] N. Atefi , M. A. Jafari , M. Roohaninasab , et al., “Evaluating the Effectiveness and Safety of Pulsed Dye Laser Alone, the Combination of Pulsed Dye Laser and Botulinum Toxin Type A, and the Combination of Pulsed Dye Laser and Triamcinolone Injection in the Treatment of Hypertrophic and Keloid Scars: A Three‐Arm Randomized Controlled Clinical Trial,” Lasers in Medical Science 40, no. 1 (2025): 92, 10.1007/s10103-025-04338-6. 39953344

[78] M. C. E. van Leeuwen , M. B. A. van der Wal , A. J. Bulstra , et al., “Intralesional Cryotherapy for Treatment of Keloid Scars: A Prospective Study,” Plastic and Reconstructive Surgery 135, no. 2 (2015): 580–589, 10.1097/prs.0000000000000911. 25626801

[79] J. Shao , M. Hu , W. Wang , et al., “Indocyanine Green Based Photodynamic Therapy for Keloids: Fundamental Investigation and Clinical Improvement,” Photodiagnosis and Photodynamic Therapy 45 (2024): 103903, 10.1016/j.pdpdt.2023.103903. 37989473

[80] X. Ou , L. Chen , C. Yu , et al., “Sandwich Therapy With Radionuclide Combined With Intralesional Injections Based on Differences in the Spatial Structure Components of Keloids,” Aesthetic Plastic Surgery 49, no. 6 (2025): 1770–1783, 10.1007/s00266-024-04442-y. 39402198 PMC11968498

[81] A. Darougheh , A. Asilian , and F. Shariati , “Intralesional Triamcinolone Alone or in Combination With 5‐Fluorouracil for the Treatment of Keloid and Hypertrophic Scars,” Clinical and Experimental Dermatology 34, no. 2 (2009): 219–223, 10.1111/j.1365-2230.2007.02631.x. 19018794

[82] K. Payapvipapong , N. Niumpradit , C. Piriyanand , S. Buranaphalin , and A. Nakakes , “The Treatment of Keloids and Hypertrophic Scars With Intralesional Bleomycin in Skin of Color,” Journal of Cosmetic Dermatology 14, no. 1 (2015): 83–90, 10.1111/jocd.12132. 25626920

[83] A. M. Erlendsson , L. K. Rosenberg , C. M. Lerche , et al., “A One‐Time Pneumatic Jet‐Injection of 5‐Fluorouracil and Triamcinolone Acetonide for Treatment of Hypertrophic Scars‐A Blinded Randomized Controlled Trial,” Lasers in Surgery and Medicine 54, no. 5 (2022): 663–671, 10.1002/lsm.23529. 35266202

[84] S. G. Chi , J. Y. Kim , W. J. Lee , et al., “Ear Keloids as a Primary Candidate for the Application of Mitomycin C After Shave Excision: In Vivo and In Vitro Study,” Dermatologic Surgery 37, no. 2 (2011): 168–175, 10.1111/j.1524-4725.2010.01846.x. 21269347

[85] M. Emad , S. Omidvari , L. Dastgheib , A. Mortazavi , and H. Ghaem , “Surgical Excision and Immediate Postoperative Radiotherapy Versus Cryotherapy and Intralesional Steroids in the Management of Keloids: A Prospective Clinical Trial,” Medical Principles and Practice 19, no. 5 (2010): 402–405, 10.1159/000316381. 20639666

[86] C. Song , H. G. Wu , H. Chang , I. H. Kim , and S. W. Ha , “Adjuvant Single‐Fraction Radiotherapy Is Safe and Effective for Intractable Keloids,” Journal of Radiation Research 55, no. 5 (2014): 912–916, 10.1093/jrr/rru025. 24801475 PMC4202283

[87] Y. Gao , W. Li , Z. Li , F. Wu , and J. Wang , “Clinical Efficacy Analysis of Botulinum Toxin Type A Combined With Superficial Radiotherapy After Chest Keloid Surgery,” Aesthetic Plastic Surgery 49, no. 22 (2025): 6333–6338, 10.1007/s00266-025-04886-w. 40369152

[88] M. Scala , P. Mereu , F. Spagnolo , et al., “The Use of Platelet‐Rich Plasma Gel in Patients With Mixed Tumour Undergoing Superficial Parotidectomy: A Randomized Study,” In Vivo 28, no. 1 (2014): 121–124. 24425846

[89] Y. M. E. Neinaa , T. A. Elsayed , D. A. Mohamed , and N. N. Elfar , “Botulinum Toxin and Platelet Rich Plasma as Innovative Therapeutic Modalities for Keloids,” Dermatologic Therapy 34, no. 3 (2021): e14900, 10.1111/dth.14900. 33605002

[90] I. K. Afridi , S. Fiaz , A. Wazir , and S. Noreen , “Intralesional Triamcinolone Alone vs. Combined Platelet‐Rich Plasma for Keloid Treatment,” Journal of the College of Physicians and Surgeons–Pakistan 35, no. 7 (2025): 922–925, 10.29271/jcpsp.2025.07.922. 40605221

[91] A. S. Kumar and K. Kamalasanan , “Drug Delivery to Optimize Angiogenesis Imbalance in Keloid: A Review,” Journal of Controlled Release 329 (2021): 1066–1076, 10.1016/j.jconrel.2020.10.035. 33091533

[92] S. Lin , G. Quan , A. Hou , et al., “Strategy for Hypertrophic Scar Therapy: Improved Delivery of Triamcinolone Acetonide Using Mechanically Robust Tip‐Concentrated Dissolving Microneedle Array,” Journal of Controlled Release 306 (2019): 69–82, 10.1016/j.jconrel.2019.05.038. 31145948

[93] S. Shaha , D. Rodrigues , and S. Mitragotri , “Locoregional Drug Delivery for Cancer Therapy: Preclinical Progress and Clinical Translation,” Journal of Controlled Release 367 (2024): 737–767, 10.1016/j.jconrel.2024.01.072. 38325716

[94] E. Vettorato , P. Volonté , U. M. Musazzi , F. Cilurzo , and A. Casiraghi , “Skin Microincision Technique to Enhance Drug Penetration for the Treatment of Keloid and Hypertrophic Scars,” International Journal of Pharmaceutics 671 (2025): 125259, 10.1016/j.ijpharm.2025.125259. 39892674

[95] B. Mbituyimana , C. F. Bukatuka , F. Qi , G. Ma , Z. Shi , and G. Yang , “Microneedle‐Mediated Drug Delivery for Scar Prevention and Treatment,” Drug Discovery Today 28, no. 11 (2023): 103801, 10.1016/j.drudis.2023.103801. 37858631

[96] Z. Sartawi , C. Blackshields , and W. Faisal , “Dissolving Microneedles: Applications and Growing Therapeutic Potential,” Journal of Controlled Release 348 (2022): 186–205, 10.1016/j.jconrel.2022.05.045. 35662577

[97] B. Y. Zhuang , F. C. Hu , X. Gao , Q. Leng , Y. Zhang , and Y. You , “Development of a Simvastatin‐Loaded Copolymer Acid‐Sensitive Nanocarrier and Its Experimental Use in the Treatment of Keloids,” Journal of Cosmetic Dermatology 24, no. 1 (2025): e16573, 10.1111/jocd.16573. 39313951 PMC11743034

[98] H. Wan , S. Wang , C. Li , et al., “LA67 Liposome‐Loaded Thermo‐Sensitive Hydrogel With Active Targeting for Efficient Treatment of Keloid via Peritumoral Injection,” Pharmaceutics 15, no. 8 (2023): 2157, 10.3390/pharmaceutics15082157. 37631371 PMC10457819

[99] W. Lv , Y. Wu , and H. Chen , “Orthogonal Upconversion Nanocarriers for Combined Photodynamic Therapy and Precisely Triggered Gene Silencing in Combating Keloids,” Journal of Controlled Release 379 (2025): 1–13, 10.1016/j.jconrel.2024.12.080. 39761860

[100] X. Han , L. Ju , C. Saengow , et al., “Nano Oxygen Chamber by Cascade Reaction for Hypoxia Mitigation and Reactive Oxygen Species Scavenging in Wound Healing,” Bioactive Materials 35 (2024): 67–81, 10.1016/j.bioactmat.2024.01.010. 38312517 PMC10835133

[101] C. H. Kuan , Y. N. Wang , E. F. Liao , et al., “Viscoelastic Hydrogel With Mechanomodulatory Tension Shielding and Time‐Dependent Immunomodulatory Effects for Scarless Healing,” Advanced Healthcare Materials 14, no. 32 (2025): e01954, 10.1002/adhm.202501954. 40833245

[102] Y. Zhong , Y. Zhang , B. Lu , et al., “Hydrogel Loaded With Components for Therapeutic Applications in Hypertrophic Scars and Keloids,” International Journal of Nanomedicine 19 (2024): 883–899, 10.2147/ijn.S448667. 38293605 PMC10824614

[103] M. Fernández‐Guarino , S. Bacci , L. A. Pérez González , M. Bermejo‐Martínez , A. Cecilia‐Matilla , and M. L. Hernández‐Bule , “The Role of Physical Therapies in Wound Healing and Assisted Scarring,” International Journal of Molecular Sciences 24, no. 8 (2023): 7487, 10.3390/ijms24087487. 37108650 PMC10144139

[104] Y. Zhao , S. Wu , Y. Cai , et al., “Integration of Finite Element Simulations With 3D Printing Technology for Personalized Chitin/PLA Microneedle‐Based Drug Delivery Systems in Thoracic Keloid Treatment,” International Journal of Biological Macromolecules 315, no. Pt 2 (2025): 144487, 10.1016/j.ijbiomac.2025.144487. 40409657

