# Supplementary material for: Global Research Landscapes in Keloid Treatment: A Bibliometric and Visual Analysis
Source: J Cosmet Dermatol. 2026 Jul 23;25(7):e71054. doi: 10.1111/jocd.71054 (PMC13396243; doi:10.1111/jocd.71054)
Supplement: Supplementary file 1 — Appendix S1: jocd71054‐sup‐0001‐AppendixS1.docx. [file JOCD-25-e71054-s001.docx]

**Supplementary Material**

The search strategy for the Science Citation Index Expanded (SCI-E) within the Web of Science Core Collection (WoSCC) database is as follows: TS=((keloid* OR cheloid* OR "keloid scar*") NOT ("hypertrophic scar*" NOT (keloid* OR cheloid*))) AND TS=((therap* OR treat* OR management OR intervention*) OR (surg* OR excis* OR resect* OR graft*) OR ("intralesional injection*" OR steroid* OR corticosteroid* OR triamcinolone OR "5-fluorouracil" OR 5-FU OR bleomycin OR verapamil OR interferon*) OR (laser* OR "carbon dioxide laser" OR "CO2 laser" OR "fractional laser" OR cryotherap* OR cryosurg* OR radiother* OR "radiation therapy" OR brachytherap* OR electron* OR "pressure therapy" OR "silicone sheet*" OR "silicone gel") OR ("botulinum toxin*" OR BTX OR "platelet rich plasma" OR PRP OR sirolimus OR mTOR OR "targeted therap*" OR "photodynamic therap*" OR PDT OR "stem cell" OR "microneedling") OR ("biomaterial*" OR "scaffold*" OR "hydrogel*" OR "film*" OR "membrane*" OR "dressing*" OR "implant*" OR "tissue engineering" OR "wound dressing" OR "drug delivery" OR "controlled release" OR "nanofiber*" OR "electrospinning" OR "alginate" OR "chitosan" OR "collagen" OR "gelatin" OR "hyaluronic acid" OR "silk fibroin" OR "extracellular matrix" OR ECM OR "decellular*" OR "3D print*"))

The search strategy for the PubMed database is as follows: ("keloid"[MeSH Terms] OR keloid* OR cheloid* OR "keloid scar") NOT "hypertrophic scarring" AND ((therap* OR treat* OR management OR intervention*) OR (surg* OR excis* OR resect* OR graft*) OR ("intralesional injection*" OR steroid* OR corticosteroid* OR triamcinolone OR "5-fluorouracil" OR 5-FU OR bleomycin OR verapamil OR interferon*) OR (laser* OR cryotherapy[MeSH Terms] OR cryotherap* OR cryosurg* OR radiotherapy[MeSH Terms] OR radiotherap* OR "radiation therapy" OR brachytherapy[MeSH Terms] OR brachytherap* OR "pressure therapy" OR "silicone sheet*" OR "silicone gel") OR ("botulinum toxins"[MeSH Terms] OR "botulinum toxin*" OR BTX OR "platelet-rich plasma"[MeSH Terms] OR "platelet rich plasma" OR PRP OR sirolimus OR mTOR OR "targeted therapy" OR "photodynamic therapy" OR PDT OR "stem cell" OR microneedling) OR ("Biocompatible Materials"[MeSH Terms] OR "Tissue Scaffolds"[MeSH Terms] OR "Hydrogels"[MeSH Terms] OR "Biological Dressings"[MeSH Terms] OR "Tissue Engineering"[MeSH Terms] OR biomaterial* OR scaffold* OR hydrogel* OR "biological dressing*" OR "wound dressing*" OR implant* OR "drug delivery" OR "controlled release" OR "nanofiber*" OR electrospinning OR "3D print*" OR alginate OR chitosan OR collagen OR gelatin OR "hyaluronic acid" OR "silk fibroin" OR "extracellular matrix" OR ECM OR decellular*))
